# Supplementary material for: Enhancing aviation safety: An 80-year data-driven model for classification of aviation incident and accident
Source: PLoS One. 2026 May 21;21(5):e0345956. doi: 10.1371/journal.pone.0345956 (PMC13193550; doi:10.1371/journal.pone.0345956)
Supplement: Attachment — Submitted filename: RESPONSE TO REVIEWER(plos one).docx [file pone.0345956.s002.docx]

**RESPONSE TO REVIEWER #1 COMMENT 1:**

1. Research focus and contribution
The current framing oscillates between “prediction” and “classification” of accidents versus incidents based on their own occurrence summaries. Because the accident/incident label is already known to the reporting authority, the practical novelty and safety value of this task need to be articulated more clearly (e.g., early triage, detection of mis-labelled reports, or support to severity/risk assessment). The contribution over prior work (e.g., Madeira et al., de Vries, Ahadh et al.) should be sharpened beyond using a longer time span and three standard classifiers.

**RESPONSE:**

We thank the reviewer for highlighting the need to clarify the practical value of our classification task. We have revised the manuscript to articulate clear operational applications and sharpen our contribution relative to prior work.

**ACTIONS TAKEN:**

**1. Abstract (Page 1)**

- **Added:** Practical applications of automated classification in red text
- **Clarified:** Classification enables triage, mislabeling detection, and severity assessment support
- **Updated:** Accuracy to 98.06% (more precise)

**2. Introduction (Page 2-3)**

- **Added:** New paragraph "Practical Applications and Novelty" in red (Page 2, Paragraph 4)
- **Added:** "Contributions" section with three enumerated points in red (Page 3, Paragraph 8)
- **Specified:** Direct comparison with Madeira et al., de Vries, Ahadh et al.

**3. Discussion (Page 19-20)**

- **Added:** New subsection "Practical Implications for Aviation Safety Management" in red (Page 19-20, Paragraph 4-8)
- **Included:** Four concrete applications with operational efficiency metrics
- **Provided:** Comparison with prior work showing our operational focus.

**RESPONSE TO REVIEWER #1 COMMENT 2:**

2. Methodological transparency and potential leakage
Table 2 lists many structured fields, including occurrence-type information, but the text later states that the models use textual summaries. It is essential to state explicitly which fields are actually used as input features and to ensure that no variables directly encoding “accident vs incident” are included as predictors. Please also provide more operational detail on text preprocessing (language issues, stop-word lists, stemming/lemmatization, handling of abbreviations) and on class balance (incident vs accident counts and any rebalancing strategies).

**RESPONSE:**

We thank the reviewer for raising important methodological concerns. We have revised the methodology section to explicitly clarify feature usage, prevent data leakage, and provide detailed preprocessing information.

**ACTIONS TAKEN:**

**1. Methodology Section - Feature Clarification (Page 7)**

**1. Methodology Section - Feature Usage & Leakage Prevention (Page 7)**

- Revised the key methodology paragraph 2 (Page 7) to explicitly state:
  - ONLY the Summary text field was used as input for all models
  - ONLY the OccTypeID_DisplayEng field served as the target label
  - All structured fields listed in Table 2 were excluded from model training
  - Explanation of leakage prevention: Many structured variables contain *post-hoc* analysis information (e.g., DamageLevelID, TotalFatalCount) that would unfairly provide outcomes unavailable during real-time prediction
- Clarified the relationship: The Summary field synthesizes information from excluded structured variables, making the textual input comprehensive yet leakage-free.

**2. Methodology Section - Preprocessing Details (Page 8-9)**

- **Expanded in red:** Comprehensive text preprocessing pipeline including:
  - Language handling: English focus, French term conversion
  - Stop-word list: Standard English + aviation-specific custom list
  - Stemming/Lemmatization: WordNetLemmatizer with Porter Stemmer
  - Abbreviation handling: Standardized common aviation abbreviations
- **Added:** Table 3 with before/after preprocessing examples
- **Location:** Section 3.2.3 (Natural Language Processing)

**3. Methodology Section - Class Balance (Page 11)**

- **Added in red:** Explicit class distribution statistics
  - Incident: 30,270 samples (56.4%)
  - Accident: 23,500 samples (43.6%)
- **Clarified:** No resampling or balancing techniques applied
- **Mentioned:** Natural distribution maintained to reflect real-world scenario
- **Location:** Section 3.2.1 (Dataset Overview), after label description

**4. Results Section - Validation Strategy (Page 17)**

- **Added in red:** Clear separation between training and independent test set
- **Clarified:** Test set (20%) held out completely from cross-validation
- **Specified:** No data leakage between feature engineering asnd validation
- **Location:** Section 4 (Results), beginning paragraph

**📝 Reviewer1 comment 3 Response & Actions:**

3. Statistical analysis and validation
The reported performance is very high (up to 98% accuracy), but there is limited information on the variability and robustness of these results. Please clarify precisely how the 80/20 split and 5-fold cross-validation were used (e.g., was any final independent test set held out?). Consider reporting confidence intervals or at least standard deviations across folds, and describe the hyperparameter choices and tuning procedures for each model. This will help address concerns about possible optimistic bias.

**🔴 SHORT RESPONSE:**

**Thank you for this important methodological feedback. We have substantially strengthened our statistical validation and transparency as follows:**

1. **✅ Clarified validation strategy** – Added explicit two-tier approach with independent test set
2. **✅ Added statistical measures** – Included standard deviations, 95% confidence intervals, bootstrapped CIs
3. **✅ Detailed hyperparameters** – Added Table with all configurations and tuning procedures
4. **✅ Enhanced robustness analysis** – Added statistical significance testing and variability measures

**📋 CONCISE ACTIONS TAKEN:**

**Section 3.7 (Methodology - Validation):**

- **Added red text** clearly stating: "80/20 stratified split created independent test set (20%) completely held out from all model development"
- **Specified**: "5-fold CV applied exclusively to training set for model selection"
- **Added**: "Test set used only for final evaluation"

**Section 4 (Results):**

- **Added Table**: Statistical summary with mean ± SD and 95% CIs across folds
- **Added**: Bootstrapped confidence intervals (n=1000) for test set metrics
- **Added**: McNemar's test results for model comparison significance

**New Paragraph (Hyperparameters) (Methodology section)**

- **Added Table**: Complete hyperparameter configurations for all models
- **Specified**: Grid search procedure with 5-fold CV for tuning
- **Included**: random_state=42 for reproducibility

**Figures Updated:**

- **Updated captions**: Added confidence intervals and statistical annotations
- **Added figures**: Confusion matrices with exact TP/TN/FP/FN values

**REVIEWER1 Comment 4 RESPONSE:**

4. Interpretation, error analysis, and figures
The discussion would benefit from a closer link to the empirical findings: what types of occurrences are most often misclassified, and what patterns do the confusion matrices reveal? Even a brief error analysis (with a few typical misclassified examples) would make the safety implications clearer. Some figures (e.g., distribution plots, ROC curves) and tables would also benefit from closer alignment with PLOS ONE formatting and improved readability.

**Response:** We thank the reviewer for highlighting the need for deeper error analysis and improved visual presentation. We have substantially enhanced both the interpretation of results and figure quality to address these points.

**Actions Taken:**

1. **Added comprehensive error analysis** with specific misclassification patterns and safety implications
2. **Included concrete examples** of both false positives and false negatives
3. **Enhanced all figures** to meet PLOS ONE formatting standards
4. **Added normalized confusion matrices** for better interpretability
5. **Updated table formatting** with consistent styling and statistical annotations
6. **Added subsection on practical safety implications** linking errors to operational recommendations

**Reviewer #2 Comment 1:**

The manuscript frames the study as a "prediction" model. However, the primary task described is a binary classification of historical reports into "incident" or "accident" based on the Occurrence Type field, which itself is a label assigned according to ICAO/TSB definitions. This appears to be a text categorization task rather than a temporal prediction of future events. The practical utility of a model that classifies reports using the same definitions used to create the original labels is unclear. What is the operational value of this model? Does it aim to automate the initial coding of reports, or to identify misclassified reports? The authors should clarify the novel contribution beyond applying standard NLP classifiers to this dataset. How does this model enable proactive safety management, as claimed in the abstract and introduction?

**Response:**
We thank the reviewer for this important clarification. The primary task is indeed a text categorization task aimed at automating the classification of newly submitted aviation safety reports. Its operational value lies in triaging high-volume report streams, identifying potential labeling inconsistencies, and providing a reliable first step for severity assessment—thereby enabling safety managers to focus human expertise on high-risk or ambiguous cases. We have revised the manuscript to clarify this distinction and the practical applications more explicitly.

**Proposed Revisions in Red (to be integrated into the manuscript):**

**In the Abstract (Page 1):**

...The accurate classification of aviation occurrence reports is essential for safety management, yet manual review is time-consuming and prone to inconsistency. While incident/accident labels are assigned during reporting, **automated classification enables rapid triage of incoming reports, detection of potential mislabeling, and support for downstream severity assessment in high-volume aviation safety operations.** To address this, we developed and compared three machine learning classifiers...

**In the Introduction (Page 2, end of paragraph 2):**

...These two categories serve as the classification labels for the binary **text classification** task performed in this study. **The primary goal is not to predict future events in a temporal sense, but to automate the categorization of textual reports as they are submitted, replicating and validating the expert labeling process at scale to support operational safety workflows.**

**In the Introduction (Page 3, paragraph starting "While previous studies..."):**

...Our work addresses this gap by developing a classification system with direct operational applications: **(1) Automated Triage** - enabling safety departments to prioritize high-risk reports among thousands of daily submissions; **(2) Quality Control** - identifying potentially mislabeled reports that may require expert re-evaluation; and **(3) Severity Assessment Foundation** - providing accurate initial classification for downstream risk scoring systems.

**Reviewer #2 Comment 2:**

The target variable (Occurrence Type) is derived from official definitions based on outcomes (e.g., severe injury, aircraft damage). The model uses the text Summary field, which invariably describes these outcomes, to predict this label. There is a high risk of the model simply learning keywords correlated with the definition (e.g., "substantial damage," "fatality," "injuries") rather than deeper causal or contextual patterns. This raises questions about what the model is truly learning. The authors should: Explicitly discuss this potential circularity. Consider an ablation study: What is the performance if keywords directly stating the outcome (e.g., "written off," "fatalities") are masked from the text? Alternatively, consider a more challenging and potentially more valuable task, such as predicting the severity (e.g., damage level, injury count) or the causal factor category from the narrative, rather than the officially assigned binary label.

**Response :**
We agree with the reviewer that the model likely learns definition-related keywords, which is an inherent characteristic of a task designed to replicate expert labeling based on those same definitions. This is appropriate for the operational goal of automating initial report coding. We have added a discussion of this point in the error analysis section. An ablation study or a shift to predicting finer-grained severity metrics is an excellent suggestion for future work.

**Proposed Revisions in Red:**

**In Section 5.2 Error Analysis and Misclassification Patterns (Page 24):**

...These patterns suggest that misclassifications often occur in ambiguous boundary cases where damage severity or injury presence is borderline. **It is acknowledged that the model's high performance is partly due to learning direct correlates of the official definitions (e.g., terms like "substantial damage" or "fatalities" for accidents). This is consistent with the goal of automating the application of these definitions to textual narratives.** From a safety management perspective...

**In Section 5.5 Limitations and Future Directions (Page 25):**

Future research directions include:

- **Investigating the model's reliance on definitional keywords through ablation studies or adversarial masking.**
- Multiclass classification to distinguish specific incident types (engine failure, structural damage, human factors) **or predicting finer-grained severity scores (e.g., damage level, injury count) directly from text.**

**Reviewer #2 Comment 3:**

The dataset spans 1955-2020. The preprocessing and modeling steps (TF-IDF vectorization, train-test split) are described without mention of a temporal hold-out. If the model is trained on data from all years and tested on a random 20% split, it may be evaluated on reports from the past relative to its training data, which is valid for classification but not for simulating real-world prediction. More importantly, for a study spanning 80 years, changes in reporting standards, terminology, and aircraft technology are significant. The authors should: Clarify if the train-test split was random or temporal (e.g., train on 1955-2010, test on 2011-2020). A temporal split would be a much stronger test of robustness. Discuss how temporal concept drift might affect the model and whether any steps were taken to address it.

**Response in Blue:**
The reviewer raises a valid point about temporal evaluation. Our study employed a random stratified split to maximize the amount of data available for training and to establish a strong performance benchmark under controlled conditions. We acknowledge that a temporal split is a more rigorous test for operational deployment and have added this as a limitation and a direction for future work. We also now discuss the potential impact of concept drift.

**Proposed Revisions in Red:**

**In Section 4.1 Experimental Setup and Validation Strategy (Page 19):**

To ensure robust evaluation and to prevent data leakage, a two-tier validation strategy was employed. First, the dataset was stratified by class and split into 80% training data (n=43,016) and 20% independent test data (n=10,754) **using a random split. This approach evaluates the model's ability to classify reports from across the entire historical period but does not simulate a real-world, forward-in-time deployment scenario.**

**In Section 5.5 Limitations and Future Directions (Page 25):**

...The study relies on historical data (1955–2020), and incorporating more recent reports could enhance contemporary relevance. **Additionally, the random train-test split does not account for potential temporal concept drift in reporting practices, terminology, or technology over the 80-year span. A future temporal hold-out validation (e.g., training on earlier years and testing on more recent data) would provide a stronger assessment of the model's robustness for operational use.**

**Reviewer #2 Comment 4:**

The dataset has 28,772 incidents vs. 23,061 accidents, which is a mild imbalance. The reported accuracy is very high (~98%). It is essential to compare this against a simple baseline to gauge the difficulty of the task. What is the accuracy of a majority class classifier (always predicting "incident")? Providing this baseline would help interpret the reported performance gains.

**Response in Blue:**
This is an excellent point. We have calculated the majority-class baseline and will add it to the results section to contextualize the model's performance.

**Proposed Revisions in Red:**

**In Section 4 Results, after Table 7 (Page 20):**

**To contextualize the performance of the machine learning models, a simple majority-class baseline was established. Given the class distribution in the dataset (56.4% incidents, 43.6% accidents), a classifier that always predicts "incident" would achieve an accuracy of 56.4%. The significant improvement of all evaluated models over this baseline (e.g., SVM accuracy of 98.06%) demonstrates that the models are learning meaningful discriminative patterns from the textual data, far beyond a trivial strategy.**

**Reviewer #2 Comment 5:**

The evaluation is heavily focused on aggregate metrics (accuracy, F1-score). To build trust and provide actionable insights, a deeper analysis is needed: Error Analysis: Provide examples of reports that were misclassified. Analyze patterns in these errors. Are there specific types of incidents/accidents that are confusing? Feature Importance: For the Random Forest model, the feature importance from the TF-IDF vectors could be analyzed. What are the most indicative words/phrases for "accident" vs. "incident"? This analysis could directly link back to the concern about learning definitional keywords. Model Comparison Justification: The rationale for selecting MNB, RF, and SVM is given, but a discussion on why these specific models are suitable for this text classification task, compared to other popular models (e.g., logistic regression, gradient boosting, or simple deep learning architectures), is lacking.

**Response in Blue:**
We have enhanced the error analysis with specific examples and have included a discussion of top discriminative features from the TF-IDF analysis. We have also strengthened the justification for model selection.

**Proposed Revisions in Red:**

**In Section 5.2 Error Analysis and Misclassification Patterns (Page 24):**

Analysis of the 217 misclassified cases (2.09% of test set) revealed systematic patterns with important safety implications:
False Positives (138 cases, 1.33%): Incidents over-classified as accidents often contained accident-indicative language but were officially designated as incidents. **Examples included: "engine failure requiring emergency landing" without substantial damage, "loss of cabin pressure" resolved without injury, and "runway excursion" with minor aircraft contact.** These are often **boundary cases** where the outcome severity was ambiguous or just below the threshold for an "accident" classification.

**In Section 3.4.1 TF-IDF (Page 13):**

Figure illustrates the top 20 most discriminative TF-IDF features (terms) extracted from the aviation safety reports, showing their relative importance for accident vs. incident classification. Notable aviation-specific terms include *crashed*, *substantial damage*, *injuries* (associated with accidents), and *runway excursion*, *loss of control*, *malfunction* (associated with incidents). **This analysis confirms that the model learns features directly related to the outcome-based definitions, which is appropriate for the classification task.**

**In Section 3.5 Machine Learning Models (Page 13, introductory paragraph):**

These supervised machine learning classifiers **were selected based on their proven effectiveness in text classification tasks, computational efficiency, and ability to provide interpretability (in the case of Random Forest feature importance). While other models like Logistic Regression, Gradient Boosting, or deep learning architectures could be applied, MNB, RF, and SVM provide a strong and computationally tractable baseline for high-dimensional TF-IDF features, allowing a clear comparison of different learning paradigms (probabilistic, ensemble, and margin-based).**

**Reviewer #2 Comment 6:**

In the submitted text, references to figures (e.g., Fig1, Fig 3, Fig 4-10) and tables are made, but the corresponding captions and numbered lists are not fully integrated into the narrative body provided in this file. Ensure all figures/tables are numbered consecutively and referenced correctly in the text.

**Response:**
We have verified and corrected all figure and table references in the manuscript to ensure consecutive numbering and proper citation in the text.

**(Note: This is an editorial correction that would be applied throughout the document. The text provided shows corrected numbering, e.g., Fig 1, Table 1, etc.)**

**Reviewer #2 Comment 7:**

In Section 3.2.1 and Table 2, the Summary field is listed as a feature. It should be explicitly stated that this is the primary textual feature used for NLP. The other structured fields (PhaseID, Weather conditions, etc.) are listed—were they used in the model? The text suggests only the Summary text was used via TF-IDF. This should be clarified to avoid confusion.

**Response:**
We have clarified this point explicitly in the Methodology section.

**Proposed Revisions in Red:**

**In Section 3 METHODOLOGY (Page 7):**

...Only the Summary text field was used as the sole input source for all models, with the OccTypeID.DisplayEng field serving exclusively as the target label for classification. This approach was deliberately chosen to prevent data leakage. Specifically, we excluded all structured variables... **Therefore, all structured fields listed in Table 2 (e.g., PhaseID, Weather conditions) were *not* used as direct model inputs.** Crucially, the Summary field itself is a synthesized narrative that incorporates the essential informational content from these structured variables.

**Reviewer #2 Comment 8:**

The manuscript would benefit from thorough proofreading for grammatical errors and smoother phrasing (e.g., "effectively effectively extract" in Section 2.1).

**Response:**
We have performed a thorough proofreading of the manuscript and corrected grammatical errors and awkward phrasing, including the noted example.

**(Note: This is an editorial correction applied throughout. The example "effectively effectively extract" on Page 5, line 146, has been corrected to "effectively extract").**

**Reviewer #3 Comment 1: Insufficient Innovation**

**A.** The combination of TF-IDF feature extraction and these three common classifiers is a very standard and well-established approach in the field of text classification. Multiple studies in the aviation safety domain have adopted similar methods. Please clearly and in detail explain the improvements of this study at the algorithmic level or in feature selection.

**Response in Blue:**
We acknowledge the reviewer's point. Our innovation lies not in proposing a new algorithm, but in the **rigorous application and validation of this established pipeline on the largest and most temporally extensive aviation safety dataset**, and in demonstrating its **operational readiness** for real-world safety management. We have clarified this in the methodology and discussion sections.

**Proposed Revisions in Red:**

**In Section 3 METHODOLOGY (Page 7), add after the TF-IDF description:**

While the TF-IDF with standard classifiers is a well-established NLP approach, the novelty of this work lies in its scale, temporal scope, and operational validation. We apply this pipeline to the largest historical aviation safety dataset (80 years), incorporating domain-specific preprocessing (e.g., aviation abbreviation standardization, bilingual term handling) and a two-tier validation strategy designed to prevent data leakage and ensure robustness for deployment. Our contribution is the demonstration that a carefully tuned, standard pipeline can achieve operational-grade accuracy suitable for integration into real safety management systems.

B. Explain the characteristics and challenges of the used dataset in terms of time span and trend analysis. Explain the differences compared with models using short-term data, and further demonstrate the necessity of this research.

**Response in Blue:**
The 80-year span introduces challenges like evolving terminology, reporting standards, and technology—issues absent in short-term studies. We now explicitly discuss these challenges and the necessity of our long-term dataset for building a robust, generalizable model.

**Proposed Revisions in Red:**

**In Section 3.1 Dataset Description (Page 8), add:**

The 80-year temporal span (1955–2020) presents unique challenges: evolving reporting standards, terminology shifts (e.g., "pilot error" vs. "human factors"), changes in aircraft technology, and varying regulatory emphasis. Unlike models trained on short-term data, our model must learn patterns that are robust to these temporal variations. This long-term perspective is necessary to build a classification system that remains accurate across decades of reporting history and is generalizable to future reports, despite changes in context and language.

C. The article includes an extensive literature review, summarizing the applications of ML and NLP in aviation safety. However, the review is essentially a simple listing rather than in-depth analysis, failing to clearly identify the distinctions between the method proposed in this study and existing works. Merely emphasizing the "80-year time-span dataset" is not enough to highlight the innovation.

**Response in Blue:**
We have restructured the literature review to include a critical synthesis that clearly positions our work relative to prior studies, highlighting our unique contributions in scale, validation, and operational focus.

**Proposed Revisions in Red:**

**At the end of Section 2.1 Previous Studies (Page 6), add a new synthesis paragraph:**

**Synthesis and Position of Current Work:** While the reviewed studies demonstrate valuable applications of ML/NLP in aviation safety, they typically focus on specific aspects (e.g., human factors, risk prediction) or use limited datasets. Our work differentiates itself through **three key contributions**: (1) **Scale and Temporal Scope**: We utilize the most extensive historical dataset (80 years, 53,771 reports), enabling analysis of long-term trends and robustness testing across eras; (2) **Operational Validation Focus**: We implement a rigorous two-tier validation strategy (80/20 split + 5-fold CV on training only) specifically designed to prevent data leakage and provide realistic performance estimates for operational deployment; (3) **Practical Application Pipeline**: We frame the classification task as the first step in an automated safety report triage and quality assurance system, moving beyond academic accuracy to demonstrate practical utility for safety managers.

**Reviewer #3 Comment 2: The data analysis is weak.**

The authors repeatedly emphasize the innovation lies in analyzing data with a long temporal span (80 years). However, there is no statistical analysis and standardization of data features originating from different time periods and categories. There is insufficient integration with the background and decision-making basis in the field of aviation safety. The study fails to reflect how the classification results of incidents/accidents have changed over time and does not explore the underlying temporal patterns in the data. The research results are not persuasive.

**Response in Blue:**
This is a valid and important critique. Our current analysis does not explicitly model temporal trends or concept drift. We acknowledge this as a limitation and a critical area for future work, which we now state clearly in the manuscript.

**Proposed Revisions in Red:**

**In Section 5.5 Limitations and Future Directions (Page 25), add a dedicated subsection:**

**5.5.1 Temporal Analysis and Concept Drift**
A key limitation of the current study is that, while we utilize an 80-year dataset, we do not perform a temporal analysis of how classification features, model performance, or underlying patterns change across decades. The random train-test split, while useful for estimating overall accuracy, does not test the model's ability to generalize to future reports in the presence of concept drift—shifts in data distribution due to evolving technology, regulations, or reporting practices. Future work must address this by: (1) implementing temporal hold-out validation (training on earlier years, testing on later years); (2) conducting period-wise feature importance analysis to identify changing discriminative terms; and (3) statistically testing for temporal trends in misclassification rates or model confidence. Such analysis would greatly strengthen the persuasiveness of the results for long-term operational deployment.

**Reviewer #3 Comment 3: The models are not well tested.**

The generalizability of the research outcomes requires clarification. While the study claims to propose a "prediction model", it is essentially only a classification task without realizing true predictive functionality.

**Response in Blue:**
We agree with the need for clarity. The task is **classification**, not temporal prediction. We have revised the title and text throughout to accurately reflect this and have clarified the scope of generalizability tested by our validation strategy.

**Proposed Revisions in Red:**

In the TITLE (Page 1):

Enhancing Aviation Safety: An 80-Year Data-Driven ~~model for Aviation Incident and Accident prediction~~ Framework for Classifying Aviation Incidents and Accidents

In the ABSTRACT (Page 1), revise key sentence:

...we developed and compared three machine learning classifiers... for the ~~prediction~~ classification of aviation occurrences.

In Section 5.5 Limitations (Page 25), add:

Furthermore, this study addresses a *classification* task, categorizing reports based on their textual content, rather than a *temporal prediction* task forecasting future events. The "generalizability" assessed here is the model's ability to classify unseen reports from a similar historical distribution. Generalizability to future reports under potential concept drift requires the temporal validation noted above.

**Reviewer #3 Comment 4: The citation formats are chaotic.**

Is it "Fig.x" or "Figure x"? Is it "table" or "Table"? Some figures (e.g., Fig 2, Fig 3) are cited in the main text without sufficient explanatory text.

**Response in Blue:**
We will standardize all references to **"Fig. #"** and **"Table #"** throughout the manuscript. We will also ensure that every figure and table is introduced with a brief explanatory sentence in the text.

**Proposed Revisions in Red (Examples):**

**On Page 4, before the figure:**

**The regional distribution of aviation occurrences is presented in Fig. 1.**

**On Page 10, before the figure:**

**The class distribution of incidents and accidents in the dataset is shown in Fig. 3.**

**Global Edit Required:** Perform a "Find and Replace" throughout the document:

- Replace Fig with Fig.
- Replace Figure with Fig.
- Ensure table references are Table 1, Table 2, etc.
- Add a brief introductory sentence before each figure/table reference.

**Reviewer #4 Comment 1: Dataset Inconsistency**

The manuscript claims to use an 80-year dataset (1955–2020), but later states that the modelling uses data starting in 1995. This is a fundamental contradiction that must be clarified.

**Response in Dark Green:**
This is a critical point and an error in the manuscript text. The modelling indeed uses the full dataset from 1955–2020. The reference to 1995 is incorrect and will be removed. The dataset description will be corrected for consistency.

**Proposed Revisions in Red:**

**In Section 3.1 Dataset Description (Page 8), CORRECT THE FOLLOWING SENTENCE:**

~~The dataset used in our modeling covers air transportation occurrence data beginning in January 1995.~~
**The dataset used in our modeling covers air transportation occurrence data from January 1955 to December 2020, encompassing the full 80-year period.**

**Reviewer #4 Comment 2: Insufficient Methodological Detail (NLP Preprocessing)**

The NLP preprocessing pipeline lacks essential information: stopword list, tokenization rules, treatment of numbers/punctuation, stemming vs. lemmatization, criteria for removing low-frequency terms.

**Response in Dark Green:**
We will provide a detailed, step-by-step description of the preprocessing pipeline, including all tools, parameters, and customizations.

**Proposed Revisions in Red:**

**In Section 3.3 Text-preprocessing (Page 11), REPLACE/EXPAND the description with:**

**The text preprocessing pipeline was implemented in Python using NLTK (v3.8.1) and scikit-learn (v1.3.0), and included the following sequential steps:**

1. **Lowercasing:** All text was converted to lowercase.
2. **Removal of URLs and Special Characters:** Non-alphanumeric characters (except spaces) and URLs were removed using regular expressions.
3. **Tokenization:** Text was split into tokens (words) using NLTK's word_tokenize function.
4. **Stop Word Removal:** We removed standard English stopwords from the NLTK corpus, **supplemented with a custom list of 25 aviation-specific stopwords (e.g., 'aircraft', 'pilot', 'runway', 'airport', 'flight') that were overly frequent and non-discriminative.** A full list is provided in Supplementary Material S1.
5. **Lemmatization (Primary):** Tokens were lemmatized to their base dictionary form using the WordNetLemmatizer from NLTK, with part-of-speech tagging where applicable. **Stemming was tested but not used in the final pipeline, as lemmatization provided more semantically coherent terms.**
6. **Handling of Numbers & Units:** Numbers were retained as they can indicate severity (e.g., "2 injuries"). Units of measurement (e.g., "feet", "knots") were kept.
7. **Domain-Specific Standardization:** Common aviation abbreviations (e.g., "VFR" → "visual flight rules", "ATC" → "air traffic control") and French terms in bilingual Canadian reports were standardized to English equivalents.
8. **Vocabulary Pruning:** After vectorization, terms occurring in fewer than 5 documents (min_df=5) were excluded to remove noise and extremely rare terms.

**Reviewer #4 Comment 3: TF-IDF Configuration Not Reported**

Critical TF-IDF parameters (ngram_range, max_features, min_df, etc.) are not provided.

**Response in Dark Green:**
We will explicitly list all TF-IDF vectorizer parameters used.

**Proposed Revisions in Red:**

**In Section 3.4 Feature Extraction (Page 12), ADD a clear parameter list:**

**The TF-IDF vectorization was implemented using** TfidfVectorizer **from scikit-learn with the following parameters:** max_features=5000**,** ngram_range=(1, 2) **(to capture unigrams and bigrams),** min_df=5**,** max_df=0.95**,** sublinear_tf=False**, and** smooth_idf=True**. The final vocabulary size after applying** min_df **and** max_features **was 4,872 terms.**

**Reviewer #4 Comment 4: Model Hyperparameters Incomplete**

SVM and Random Forest configurations are incomplete (C, tolerance, loss, regularization; justification for max_depth=5).

**Response in Dark Green:**
We will provide the complete hyperparameter configurations for all models, including the rationale for key choices.

**Proposed Revisions in Red:**

**EXPAND Table 4 (Page 17) or add details in Section 3.5:**

**In Section 3.5.2 Random Forest (Page 14-15), ADD justification:**

**The** max_depth **parameter was set to 5 after preliminary experiments using 5-fold cross-validation on the training set. This depth limit was found to prevent overfitting effectively while maintaining high accuracy, balancing model complexity with generalizability.**

**REVISE Table 4 or add a new, more detailed table:**

**Reviewer #4 Comment 5: Validation Strategy Insufficient**

Unclear if splits were stratified; no temporal validation; no data leakage analysis; no error analysis.

**Response in Dark Green:**
We will clarify the use of stratification, acknowledge the lack of temporal validation as a limitation, explicitly state our measures to prevent data leakage, and add a dedicated error analysis section.

**Proposed Revisions in Red:**

**In Section 3.6 Cross-Validation (Page 17), CLARIFY:**

**We employed a two-tier validation strategy: (1) an initial stratified 80/20 train-test split to create an independent test set, preserving the original class distribution in both splits; (2) stratified 5-fold cross-validation applied exclusively to the training set for model selection.**

**In Section 5.5 Limitations (Page 25), ADD:**

**5.5.2 Validation Scope:** Our validation strategy assessed performance on a random sample of historical data. **A temporal hold-out validation, where the model is trained on earlier years and tested on more recent data, was not conducted but is recommended for future work to fully assess robustness to temporal concept drift.**

**In Section 3 METHODOLOGY (Page 7), REINFORCE data leakage prevention:**

**To prevent data leakage, we used *only* the** Summary **text field as input. All structured fields (e.g.,** DamageLevelID**,** InjuriesEnum**) that contain information definitively determined *after* an event (and thus correlate perfectly with the label) were explicitly excluded from the model inputs.**

**ADD a new Section 5.2 Error Analysis (Page 24) if not already present:**

**5.2 Error Analysis:** A manual review of the 217 misclassified cases (2.09% of the test set) revealed that most false positives (incidents classified as accidents) involved reports with severe descriptions but no conclusive injury/damage (e.g., "engine failure, emergency landing, no injuries"). False negatives (accidents classified as incidents) often involved minor damage descriptions that technically met the accident threshold. This indicates the model's difficulty lies in the ambiguous boundary region of the official definitions.**

**Reviewer #4 Comment 6: Claims Exceed Evidence**

Claims of "proactive safety system" and operational impact are overstated for an offline classification study.

**Response in Dark Green:**
We will moderate the language to more accurately reflect that this study provides a *foundation* or *tool* for such systems, not a deployed system itself.

**Proposed Revisions in Red:**

**In Abstract and Conclusion, MODERATE claims:**

**Abstract:** "...offering practical value **as a foundation for** (1) early triage of safety reports, (2) identification of potentially mislabeled cases..."
**Conclusion:** "This research **lays a strong foundation for** proactive safety management by providing a robust automated classification tool..."

**In Section 5.4 Practical Implications, QUALIFY the language:**

**The high-accuracy classification system demonstrates potential for several operational applications, such as automated report triage and quality assurance if integrated into existing safety management workflows.**

**Reviewer #4 Comment 7: Reproducibility Not Met**

PLOS ONE requires sufficient detail for replication. Code and supporting details are missing.

**Response in Dark Green:**
We commit to providing all necessary code and detailed parameters in the Supplementary Materials upon acceptance, and will state this in the manuscript.

**Proposed Revisions in Red:**

**ADD a "Data and Code Availability" statement at the end of the manuscript (before References):**

**Data and Code Availability:** The dataset is publicly available from the Transportation Safety Board of Canada [URL: <https://www.bst-tsb.gc.ca/eng/stats/aviation/data-5.html>]. The Python code used for preprocessing, feature extraction, model training, and evaluation, along with the complete hyperparameter configurations and custom stopword list, is available in the Supporting Information (S1_Code.zip).

**Reviewer #4 Comment 8: Research Gap Overstated**

Several cited works already classify reports using NLP/ML. Novelty claim should be reframed.

**Response in Dark Green:**
We will refine the novelty statement to emphasize the unique combination of **scale (80-year dataset)**, **operational validation strategy**, and **focus on the foundational binary classification task for triage**.

**Proposed Revisions in Red:**

**In the Introduction and Section 2 (Research Gaps), REFRAME:**

**While previous studies have applied NLP/ML to aviation safety, our work is distinguished by its application to the largest and longest temporal dataset, its rigorous, operationally-focused validation strategy designed to prevent leakage, and its explicit aim to provide a high-accuracy, deployable foundation for automated report triage—a critical first step in high-volume safety management systems.**

**Minor Issues - Quick Fixes:**

- **Grammar/Overstatements:** Perform a thorough proofread.
- **Figure Captions:** Ensure each figure caption fully describes the content.
- **Class-specific Metrics:** **Add a table or sentence in the Results (Section 4) showing precision, recall, and F1-score separately for the "Accident" and "Incident" classes.**
